# Prevalence, risk factors, and outcomes of acute kidney injury in a pediatric cardiac intensive care unit: A cross‐sectional study

**DOI:** 10.1002/hsr2.1791

**Published:** 2024-01-04

**Authors:** Zahra Esmaeili, Fahimeh Asgarian, Ehsan Aghaei Moghadam, Amirali Khosravi, Behdad Gharib

**Affiliations:** ^1^ School of Medicine Tehran University of Medical Sciences Tehran Iran; ^2^ Children's Medical Center Tehran University of Medical Sciences Tehran Iran

**Keywords:** acute kidney injury, cardiac intensive care unit, critical illness, intensive care units, KDIGO, pediatric intensive care unit

## Abstract

**Background and Aims:**

Acute kidney injury (AKI) is a common complication in pediatric cardiac intensive care unit (CICU). This study aims to identify the prevalence, risk factors, and outcomes of AKI in pediatrics admitted to a CICU unit of a tertiary hospital.

**Methods:**

We retrospectively gathered the data of 253 randomly selected patients admitted to the CICU unit from March 2018 to March 2022. Data were collected from EHRs. We used the Kidney Disease Improving Global Outcomes (KDIGO) criteria for identifying AKI in patients.

**Results:**

Overall, AKI prevalence was 22.9% in our population. In the multivariable analysis, vancomycin intake (odds ratio [OR]: 2.109, 95% confidence interval [CI]: 1.15–3.84), angiography (OR: 4.38, 95% CI: 1.28–14.93), and mechanical ventilation (OR: 2.08, 95% CI: 1.02–4.23) were independent risk factors of AKI development and patients with AKI had a higher in‐hospital mortality rate (OR: 5.81, 95% CI: 2.55–13.19), higher need for cardiopulmonary resuscitation (OR: 3.08, 95% CI: 1.17–8.09), and longer ICU length of stay (OR: 6.49, 95% CI: 3.31–9.67). Furthermore, furosemide administration was associated with lower risk of developing AKI (OR: 0.52, 95% CI: 0.27–0.97).

**Conclusion:**

AKI is common and is associated with worse outcomes in patients with congenital heart disease. Our results emphasize the importance of early identification and monitoring of AKI in the pediatric CICU setting.

## 1 INTRODUCTION

The survival of patients with congenital heart diseases has increased dramatically due to recent advances in cardiac surgery, yet morbidity rates remain high.^1^, ^2^ Acute kidney injury (AKI), defined as an abrupt decline in renal function, is a common complication in critically ill patients admitted to the pediatric cardiac intensive care unit (PCICU).^3^, ^4^ Studies show a wide range of AKI prevalence in patients, from 4.5% in developed countries to 58% in developing countries^5^, ^6^, ^7^, ^8^; different AKI definitions can also contribute to this variation.^9^, ^10^, ^11^, ^12^ The Kidney Disease Improving Global Outcomes (KDIGO), provides the most harmonized definition for AKI diagnosis, staging, and management.^13^

Children undergoing cardiac surgery are at risk for developing cardiorenal syndrome (CRS). CRS is a condition in which diminished cardiac function in conjunction with renal dysfunction can lead to decreased urine output and fluid retention, impairing the function of both systems.^1^, ^14^ Several other factors lead to renal dysfunction in these patients. Nephrotoxic drugs, infections, contrast agents, and excessive fluid administration can worsen CRS.^3^, ^15^ Developing AKI in cardiac patients, even in minor degrees, can cause adverse complications, such as higher mortality rates, prolonged hospital admission, and hemodynamic instability.^16^, ^17^, ^18^ In addition to in‐hospital complications, studies have shown an association between AKI and the long‐term risk of chronic kidney disease (CKD) in children.^15^, ^19^

To the authors' knowledge, most of the published studies have assessed the AKI incidence, risk factors, and outcomes in patients after cardiac surgery^4^, ^19^, ^20^, ^21^, ^22^, ^23^ in the CICU, and investigating this issue in the nonsurgical population is not remarkable. This retrospective observational study aims to examine the prevalence of AKI in CICU patients with congenital or acquired heart disease and to determine the associated factors that may identify patients at high risk for AKI and the in‐hospital outcomes of AKI in patients. It can help future prospective experiments examine whether modifying these factors can help reduce the burden of AKI in patients.

## 2 METHOD

### 2.1 Study design and setting

This observational study was conducted in October 2022 at Children's Medical Center Hospital, a tertiary center in Tehran, Iran. We retrospectively gathered the data from electronic health records (EHRs) among all patients admitted to the PCICU from March 2018 to March 2022. The PCICU ward in this hospital serves critically ill patients with primary or secondary cardiac disease. Immediately postoperative patients are admitted and cared for in a separate intensive care unit. To reduce the risk of bias, only the data from the first admission episode was included in the analysis. We conducted this study in accordance with the Strengthening the Reporting of Observational Studies in Epidemiology (STROBE) guidelines to enhance the transparency and completeness of our research. Tehran University of Medical Sciences (TUMS) research ethics committee approved the conduct of this study without explicit consent from the participants (ethical code: IR.TUMS.CHMC.REC.1400.027).

### 2.2 Exclusion criteria

2.2.1

Patients with the following factors were excluded from the study: Age ≤30 days old or ≥18 years old, duration of admission less than 3 days, incomplete records regarding the diagnosis of AKI and measured outcomes, and presence of an underlying known renal disease or CKD.

### 2.3 Data extraction: Definition of variables

The following data were recorded: Basic demographic data (age, sex, weight), type of congenital heart disease, fluid limitation, administered drugs (furosemide, vancomycin, aminoglycoside), use of mechanical ventilation, and angiography during the admission. In‐hospital mortality, ICU length of stay (LOS), and the need for cardiopulmonary resuscitation (CPR) were recorded as outcome variables.

We classified the type of heart disease into three subgroups. Cyanotic heart disease includes tetralogy of Fallot, pulmonary atresia, single ventricle, Ebstein anomaly, and total anomalous pulmonary venous return. Patients with atrial septal defect, ventricular septal defect, and patent ductus arteriosus were considered as acyanotic heart disease and nonstructural heart disease, including patients with myocarditis, cardiomyopathy, and arrhythmia.

We calculated the fluid maintenance need based on the Holliday–Segar method^24^, ^25^; patients who did not get the calculated daily fluid maintenance were categorized in the limited fluid intake group. Furosemide, aminoglycoside, and vancomycin intake were considered positive in the AKI group only if they were administered within 7 days of developing AKI.^26^, ^27^, ^28^ We retrieved the angiographic data of all patients who had received an angiographic study during the current admission from their medical records.

### 2.4 AKI definition

AKI was defined based on the KDIGO criteria.^13^ Patients with increased serum creatinine (sCr) ≥ 0.3 mg/dL within 48 h or increased sCr ≥ 1.5 times baseline within the previous 7 days were considered as AKI based on these criteria. We used only the sCr for the classification of AKI, as this was a retrospective study, and detailed urine output measurements could not be obtained. Baseline serum creatinine (mg/dL) was the lowest serum creatinine level measured during hospitalization before developing AKI.

### 2.5 Outcomes

The primary outcome of the study was to investigate the incidence of AKI according to the KDIGO criteria and to identify the probable associating factors of developing AKI in these patients. The secondary outcome was comparing the mortality rate, need for CPR, and CICU LOS between the AKI and non‐AKI groups.

### 2.6 Study size

During the mentioned period of time, 1900 patients were admitted to the PCICU unit. Sample size calculations were performed using the power analysis method, considering a 95% confidence interval, 80% statistical power, and an effect size of 5%; we reached the number of 247 patients. Using the random number method, 253 patients were selected for further analysis. The patients not diagnosed with AKI based on the KDIGO criteria served as controls. Patients were followed up until discharge or death.

### 2.7 Statistical analysis

Continuous variables with normal distribution were described by mean with standard seviation (SD), and those without a normal distribution were described as medians with interquartile range (IQR). Categorical variables were presented with absolute and relative frequencies (%). To compare the baseline characteristics of the study population and identify the risk factors among the AKI group, univariate analysis was performed using the *χ* ^2^ test and Fisher's exact test for the categorical variables and Mann–Whitney *U*‐test for the continuous variables (age, sex, ICU LOS). Statistical significance was considered as *p* < 0.05. The odds ratio (OR) and 95% confidence interval (CI) were calculated to evaluate the difference between the two groups. Multivariable logistic regression and multiple regression analyses were performed to compare the outcome variables (mortality, CICU LOS, and need for CPR) and identify the independent risk factors for AKI development. Confounding factors were identified as clinically essential variables, which lowered the OR to ≥10% in multivariable analysis.^29^ All statistical tests were conducted as two‐sided analyses using IBM SPSS Statistics version 26.

## 3 RESULTS

### 3.1 Population

During the study period, 1900 patients were admitted to the PCICU unit. Two hundred fifty‐three patients were randomly selected for final analysis. Fifty‐eight patients (22.9%) were classified as AKI. Those who did not develop AKI served as controls (i.e., 195 controls).

### 3.2 Baseline characteristics

The baseline characteristics of patients in both groups and their underlying heart disease are demonstrated in Table 1. The median age of the AKI group was 0.37 (IQR: 0.08–3.06 years) compared to the non‐AKI groups, with a median age of 1 (IQR: 0.33– 4.33 years), and 50% of the AKI group patients were male. Patients in the AKI group had a lower weight with a median of 5.85 (IQR: 4–10 kg). Baseline serum creatinine in the AKI group was lower compared to our non‐AKI group (mean sCr of 0.47 compared to 0.53 mg/dL).

**Table 1:** Basic characteristics of patients in both groups.

	Total (*N* = 253)	AKI (*N* = 58)	Non‐AKI (*N* = 195)
Age (years)	0.75 (0.25–4.12)	0.37 (0.08–3.06)	1 (0.33–4.33)
Sex (male), *n* (%)	139 (54.9)	29 (50)	110 (56.4)
Weight (kg)	6.8 (4.4–13)	5.85 (4–10)	7 (4.5–14)
Congenital heart disease			
Cyanotic, *n* (%)	94 (37.2)	19 (32.8)	75 (38.5)
Acyanotic, *n* (%)	106 (41.9)	26 (44.8)	80 (41)
Others, *n* (%)	53 (20.9)	13 (22.4)	40 (20.5)

Abbreviation: AKI, acute kidney Injury.

### 3.3 AKI risk factors

Univariable analysis for identifying risk factors for AKI development is presented in Table 2. Lower age and weight, vancomycin administration, mechanical ventilation, and angiography were associated with a higher risk of AKI development in patients (*p* < 0.05). Patients who had furosemide intake had a lower risk of developing AKI. Regarding the aminoglycoside administration, there was no statistically significant difference between the two groups. (*p* > 0.05).

**Table 2:** Univariate analysis for AKI risk factors.

	Total (*N* = 253)	AKI (*N* = 58)	Non‐AKI (*N* = 195)	*p* Value
Age (years)	0.75 (0.25–4.12)	0.37 (0.08–3.06)	1 (0.33–4.33)	0.02
Sex (male), *n* (%)	139 (54.9)	29 (50)	110 (56.4)	0.38
Weight (kg)	6.8 (4.4–13)	5.85 (4–10)	7 (4.5–14)	0.04
Congenital heart disease				0.67
Cyanotic, *n* (%)	94 (37.2)	19 (32.8)	75 (38.5)	
Acyanotic, *n* (%)	106 (41.9)	26 (44.8)	80 (41)	
Others, *n* (%)	53 (20.9)	13 (22.4)	40 (20.5)	
Limited fluid intake, *n* (%)	67 (26.5)	20 (34.5)	47 (24.1)	0.11
Furosemide intake, *n* (%)	184 (72.7)	36 (62.1)	148 (75.9)	0.03
Vancomycin intake, *n* (%)	84 (33.2)	27 (46.6)	57 (29.2)	0.01
Aminoglycoside intake, *n* (%)	37 (14.6)	9 (15.5)	28 (14.4)	0.82
Angiography, *n* (%)	11 (4.3)	6 (10.3)	5 (2.6)	0.02
Mechanical ventilation, *n* (%)	43 (17)	15 (25.9)	28 (14.4)	0.04

Abbreviation: AKI, acute kidney injury.

To identify the independent risk factors of developing AKI, multivariable logistic regression analysis was done (Table 3). In the multivariable analysis after adjustment for identified confounding factors, only vancomycin administration (OR: 2.109, 95% CI: 1.15–3.84), angiography (OR: 4.38, 95% CI: 1.28–14.93), and mechanical ventilation (OR: 2.08, 95% CI: 1.02–4.23) were associated with developing AKI in patients (*p* < 0.05). Furosemide intake had a protective effect on developing AKI (OR: 0.52, 95% CI: 0.27–0.97).

**Table 3:** Independent risk factors of AKI, multivariable logistic regression analysis.

Risk factor	OR, 95% CI	*p* Value
Age	1.06 (0.91–1.22)	0.42
Weight	0.97 (0.94–1.01)	0.16
Sex	1.29 (0.71–2.32)	0.39
Congenital heart disease		
Cyanotic	0.77 (0.41–1.44)	0.43
Acyanotic	1.16 (0.64–2.10)	0.60
Others	1.11 (0.55–2.27)	0.75
Limited fluid intake	1.86 (0.97–3.56)	0.06
Furosemide intake	0.52 (0.27–0.97)	0.04
Vancomycin intake	2.109 (1.15–3.84)	0.02
Aminoglycoside intake	1.09 (0.48–2.47)	0.82
Angiography	4.38 (1.28–14.93)	0.02
Mechanical Ventilation	2.08 (1.02–4.23)	0.04

Abbreviations: AKI, acute kidney injury; CI, confidence interval; OR, odds ratio.

### 3.4 Outcome analysis

Mortality, need for CPR, and ICU LOS were compared between the two groups. Overall mortality and need for CPR in our population were 11.1% and 9.9%, respectively, significantly higher in our AKI population (27.6% vs. 6.2% *p* < 0.001, 20.7% vs. 6.7% *p* = 0.002). ICU LOS had a median of 6 days in our total population, and it was significantly higher in our AKI group (9 [5–16.75] vs. 6 [3–9] days, *p* = 0.01). After multivariable analysis, mortality (OR: 5.81, 95% CI: 2.55–13.19), need for CPR (OR: 3.08, 95% CI: 1.17– 8.09), and ICU LOS (OR: 6.49, 95% CI: 3.31–9.67) remained higher in our AKI group (Table 4).

**Table 4:** Comparison of outcome variables between groups.

	Total	AKI (*N* = 58)	Non‐AKI (*N* = 195)	*p* Value	Adjusted OR^a^
In‐hospital mortality, *n* (%)	28 (11.1)	16 (27.6)	12 (6.2)	<0.001	5.81 95% CI (2.55– 13.19)
Cardiopulmonary resuscitation, *n* (%)	25 (9.9)	12 (20.7)	13 (6.7)	0.002	3.08 95% CI (1.17– 8.09)
ICU LOS					
Mean (SD)	9.53 (10.94)	14.5 (16.24)	8.06 (0.60)	0.01	6.49 95% CI (3.31–9.67)
Median (IQR)	6 (4–10)	9 (5–16.75)	6 (3–9)		

Abbreviations: AKI, acute kidney injury; CI, confidence interval; ICU LOS, intensive care unit length of stay; IQR, interquartile range; OR, odds ratio.

^a^ OR in multivariable analysis.

## 4 DISCUSSION

The prevalence of AKI in our study was 22.9%. Patients in the AKI group were younger and had a lower weight compared to our non‐AKI group. Vancomycin administration, mechanical ventilation, and angiography were independent risk factors for AKI development. Furosemide administration had a protective effect on developing AKI. Patients in the AKI group had a higher mortality rate, higher need for CPR, and longer ICU LOS.

### 4.1 AKI prevalence

The prevalence of AKI in our study population, a tertiary pediatric hospital in Iran, was 22.9%. In previous studies, the prevalence of AKI in the pediatric intensive care unit varies from 4.5% to 58% based on the diagnostic criteria used and the study population.^5^, ^6^, ^7^, ^8^ Published studies show a higher prevalence of AKI in developing countries compared to developed countries.^5^, ^7^, ^30^, ^31^ Sutherland et al. compared the incidence of AKI in their ICU population based on three different criteria: Pediatric RIFLE, AKI Network (AKIN), and KDIGO. Based on the three criteria, the AKI incidence in this study was 51.1%, 37.3%, and 40.3%, respectively.^11^ Sampaio et al. have evaluated the incidence and risk factors of AKI after cardiac surgery in patients based on the three previous criteria. The incidence of AKI was 15%, 51%, and 19%, respectively, based on the RIFLE, AKIN, and KDIGO criteria. Moreover, it is also concluded that the KDIGO criteria have the most significant prognostic power for patient outcome prediction.^10^

### 4.2 Outcome

In our study, AKI was an independent risk factor for developing mortality and the need for CPR. Similar to our study, previous studies have found a higher mortality rate in patients with AKI.^20^, ^21^, ^31^, ^32^, ^33^, ^34^ Patients with AKI often are critically ill and have multiorgan damage, which can lead to higher mortality rates in this population. In some other studies, AKI was not found to be an independent risk factor for mortality.^5^, ^35^ Naik and colleagues found the need for mechanical ventilation and multiorgan failure as independent predictors of mortality.^5^ Although several studies have assessed the risk of AKI development after a successful CPR,^36^, ^37^, ^38^ to the authors' knowledge, the need for CPR after developing AKI has not been investigated. However, as mentioned above, substantial evidence supports the higher mortality rates in AKI patients, which builds the rationale for a higher need for CPR in such patients.

ICU LOS was higher among the AKI group in our multivariable analysis (OR: 6.49, 95% CI: 3.31–9.67), which aligns with previous studies findings.^7^, ^18^, ^30^, ^39^ Zappitelli et al. found that AKI, whether defined as a ≥50% rise in sCr or ≥25% rise in sCr, was independently associated with longer ICU LOS. This suggests that even a mild renal function disruption can cause poorer patient outcomes.^18^ In the study of Schenider et al., the presence of AKI at admission or developing at any time during the ICU course was accompanied by longer ICU LOS in patients. Although there was no significant difference between LOS in patients with any stage of AKI based on the RIFLE criteria.^30^ However, reverse causality may have an effect, as patients with higher ICU LOS are exposed to more nephrotoxic drugs or may experience periods of hypotension which may contribute to the development of AKI.^39^, ^40^

### 4.3 AKI risk factors

We found that vancomycin administration, mechanical ventilation, and angiography were independent risk factors of AKI development. Several studies have assessed risk factors for developing AKI in pediatrics admitted to ICU.^35^, ^41^, ^42^, ^43^ Mehta et al. found that patients with younger age, shock, sepsis, and need for mechanical ventilation were at higher risk of developing AKI.^35^ Patients at younger ages may have a greater risk of developing AKI, probably due to the immature renal function of younger patients and lower creatinine clearance.^35^, ^41^ Gupta et al. identified using vasoactive drugs, nephrotoxic drugs, and mechanical ventilation as risk factors for developing AKI.^42^ It could be mainly because patients dependent on mechanical ventilation are more critically ill and experience multiorgan failure and sepsis, raising the need for antibiotics with potential nephrotoxic effects such as vancomycin; the association of these factors can have a synergistic effect on developing AKI. Similar to previous reports,^44^, ^45^, ^46^ we found that patients with prior angiography within their admission had a higher risk of AKI development. Several factors during the angiography process can affect the risk of developing AKI, including the hydration status, age, comorbidities such as heart failure, intraoperative bleeding, hypotension, duration of the procedure, and use of nephrotoxic contrast media.^47^, ^48^

Recent studies have controversial opinions regarding the effect of furosemide administration on developing AKI.^49^, ^50^, ^51^, ^52^ Our study demonstrated that administering furosemide could significantly decrease the rate of AKI in patients. Our finding aligns with the study of Zhao et al., who reported a recovery in renal function and short‐term survival of patients with AKI.^51^ Based on previous evidence, furosemide prevents kidney injury by reducing the rate of oxygen consumption and increasing the renal blood flow.^53^, ^54^ Conversely, several studies did not authenticate furosemide administration for preventing renal dysfunction.^55^, ^56^ It is necessary to investigate further the effect of furosemide on renal function in more extensive and prospective trials.

### 4.4 Limitation

Our study had some limitations. It was a retrospective study in the Children's Medical Center Hospital CICU unit, a referral center, so the results cannot be applied to the general pediatric population. We used the existing medical records, so there was some lack of data for detecting AKI. Urine output was not included in our diagnostic criteria for AKI, but it is an important marker of AKI severity. Using urine output for detecting AKI could affect the observed prevalence of AKI in our population. This study did not include the type and volume of contrast media used for the angiography, which could influence renal dysfunction. Our study was an observational retrospective study, so it cannot build a definite cause‐and‐effect relationship between the detected high‐risk variables and AKI development.

## 5 CONCLUSION

AKI is common among patients admitted to CICU and is associated with a higher mortality rate, need for CPR, and longer ICU LOS. AKI was higher among patients with mechanical ventilation, vancomycin administration, and prior angiography. These findings can help healthcare professionals to identify at‐risk patients and prevent the development of adverse outcomes. Further research is warranted to improve outcomes and optimize care for these vulnerable patients.

## AUTHOR CONTRIBUTIONS

**Zahra Esmaeili**: Conceptualization; formal analysis; investigation; methodology; software; writing—original draft. **Fahimeh Asgarian**: Conceptualization; methodology; supervision; writing—review and editing. **Ehsan Aghaei Moghadam**: Conceptualization; methodology; project administration; supervision; validation; writing—review and editing. **Amirali Khosravi**: Conceptualization; data curation; formal analysis; investigation; software. **Behdad Gharib**: Conceptualization; methodology; project administration; supervision; validation; writing—review and editing. All authors have read and approved the final version of the manuscript. Dr. Behdad Gharib had full access to all of the data in this study and takes complete responsibility for the integrity of the data and the accuracy of the data analysis.

## CONFLICT OF INTEREST STATEMENT

The authors declare no conflict of interest.

## TRANSPARENCY STATEMENT

The lead author Behdad Gharib affirms that this manuscript is an honest, accurate, and transparent account of the study being reported; that no important aspects of the study have been omitted; and that any discrepancies from the study as planned (and, if relevant, registered) have been explained.

## Data Availability

The data that support the findings of this study are openly available in the Mendeley Data repository at https://doi.org/10.17632/2665twvr5g.1.
